# Current status of perimesencephalic non-aneurysmal subarachnoid hemorrhage

**DOI:** 10.3389/fneur.2022.960702

**Published:** 2022-09-01

**Authors:** Kun Hou, Jinlu Yu

**Affiliations:** Department of Neurosurgery, The First Hospital of Jilin University, Jilin, China

**Keywords:** perimesencephalic non-aneurysmal subarachnoid hemorrhage, etiology, complication, prognosis, review

## Abstract

Perimesencephalic nonaneurysmal subarachnoid hemorrhage (PNSAH) is a distinctive disease, representing SAH centered in perimesencephalic cisterns, with negative angiography findings. In recent years, the number of patients with PNSAH has increased significantly; however, the knowledge of PNSAH is insufficient. Therefore, we performed a review of the literature from a PubMed search and recounted our understanding of PNSAH. In this review, we summarized that current high-resolution computed tomography angiography is an acceptable replacement for digital subtraction angiography to rule out aneurysms in PNSAH with strict criteria. The current hypothesis about the etiology of PNSAH is that there is deep vein rupture from aberrant venous anatomy and increased intracranial venous pressure. PNSAH is associated with mild symptoms and lower rates of hydrocephalus and symptomatic vasospasm. For PNSAH, conservative treatment has been the mainstream treatment. PNSAH has a benign clinical course and an excellent prognosis; in long-term follow-up, re-bleeding and death were uncommon.

## Introduction

**Figure 1:**
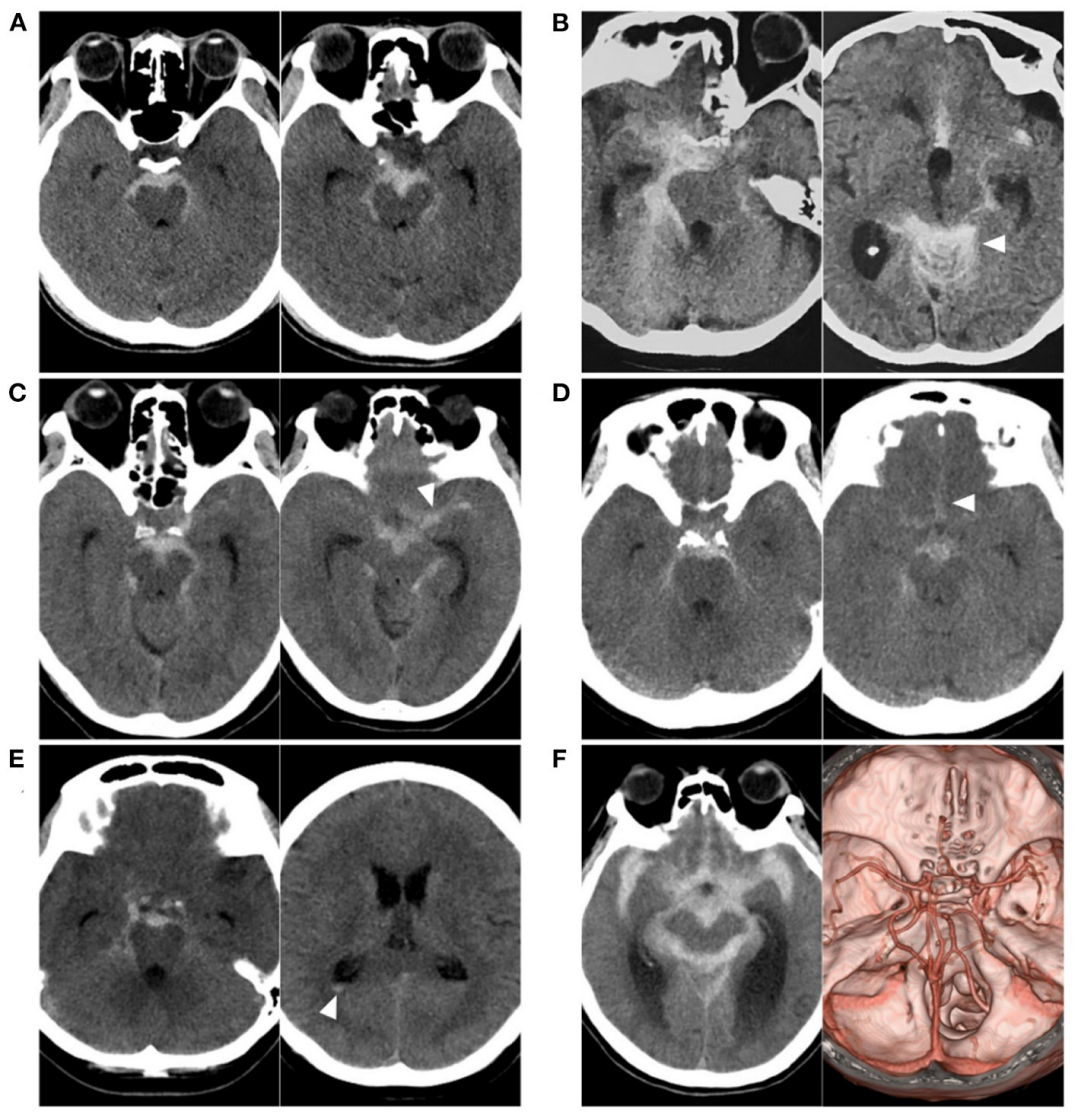
Various SAH with negative angiography. **(A)** CT showing a PNSAH only with focal pre-pontine hemorrhage. **(B)** CT showing a PNSAH with involvement of quadrigeminal cistern (arrowhead). **(C)** CT showing a PNSAH extended to the basal part of the Sylvian fissure (arrowhead). **(D)** CT showing a PNSAH extended to the posterior part of the interhemispheric fissure (arrowhead). **(E)** CT showing a PNSAH with intraventricular sedimented blood (arrowhead). **(F)** Left: CT showing a diffuse non-PNSAH; Right: the intracranial aneurysm was not found on CTA; the drainage pattern of the deep vein around vein of Galen was normal. CT, computed tomography; CTA, computed tomography angiography; PNSAH, perimesencephalic non-aneurysmal subarachnoid hemorrhage; SAH, subarachnoid hemorrhage.

In up to 15% of patients with spontaneous subarachnoid hemorrhage (SAH), including perimesencephalic non-aneurysmal SAH (PNSAH) (Figures 1A–E), diffuse non-aneurysmal SAH (Figure 1F), and sulcal SAH (1–3), no aneurysm can be identified as a source of hemorrhage. PNSAH is distinctive; it was first described by van Gijn et al. (4) in 1985, representing SAH centered in focal perimesencephalic cisterns. The incidence can be estimated to be ≈0.5 per 100.000 person-years, representing 6.8% of spontaneous SAHs (5). Since 2000, the number of patients with PNSAH has increased significantly (6).

Not enough is known about PNSAH; for instance, is computed tomography angiography (CTA) sufficient for diagnosing PNSAH? What is the etiology of PNSAH? Why does PNSAH have a benign clinical course and excellent prognosis? Therefore, it is necessary to conduct a review of the literature and recount our understanding of PNSAH. Additionally, we provide important images and educational cases to increase reading interest. These images and cases are from our hospital, and there are no copyright disputes.

## Definition and variant

### Definition

In PNSAH, the angiographic results of brain vessels are negative, and no definite SAH causes can be identified. PNSAH was commonly defined using the guidelines by Rinkel et al. (7) in 1991 based on computed tomography (CT) or magnetic resonance imaging (MRI). In 2016, Wallace et al. expanded the definition (8). When diagnosing PNSAH, CT or MRI should be obtained as early as possible, as beyond 72 hours, the SAH will redistribute and alter the hemorrhagic pattern (9, 10).

**Figure 2:**
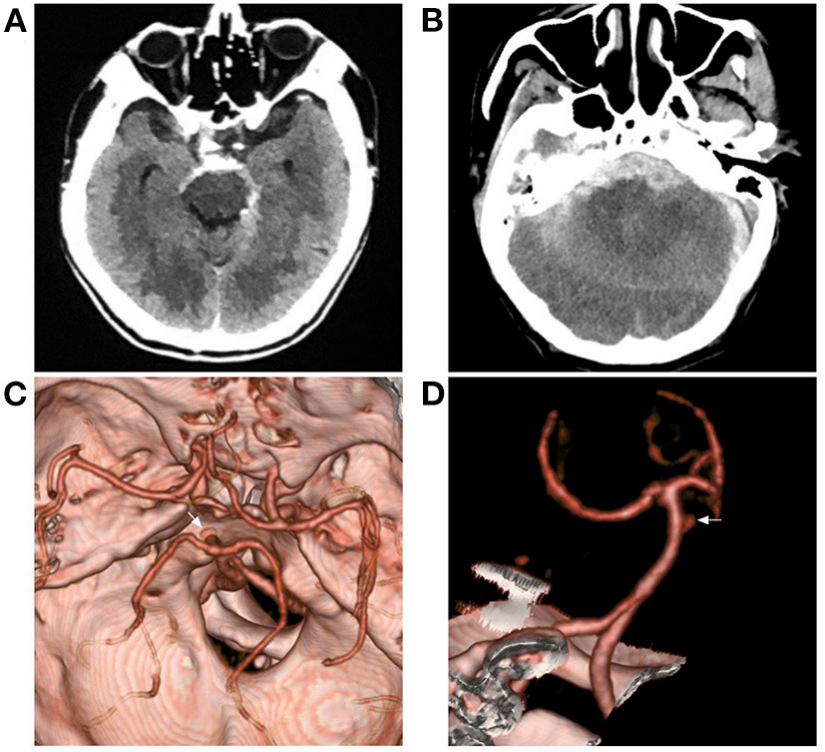
Aneurysmal SAH with perimesencephalic bleeding pattern. **(A)** Immediate CT of the onset showing SAH with typical perimesencephalic bleeding pattern. **(B)** Repeated CT 6 h after the onset showing a second SAH with the thick hemorrhage in front of the brainstem. **(C,D)** CTA showing an aneurysm of the basilar artery (arrows). CT, computed tomography; CTA, computed tomography angiography; SAH, subarachnoid hemorrhage.

**Figure 3:**
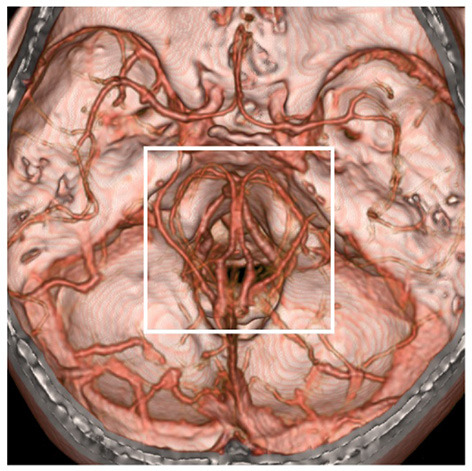
Region of possible etiology of PNSAH. By extending the time of image acquisition of computed tomography angiography, both arteries and deep veins are detected simultaneously. The possible etiology of PNSAH should focus on the frame region. PNSAH, perimesencephalic non-aneurysmal subarachnoid hemorrhage.

The criteria for PNSAH are as follows: (1) the SAH epicenter is immediately anterior to the midbrain and/or pons with possible extension to the ambient or quadrigeminal cisterns (Figures 1A,B); (2) it is acceptable for the SAH to extend to the posterior part of the anterior interhemispheric fissure and the basal part of the Sylvian fissure (Figures 1C,D); (3) it is acceptable that there are small amounts of sedimented blood in the lateral ventricle occipital horn and fourth ventricle (Figure 1E), but frank intraventricular blood and intracerebral hematoma are not acceptable; and (4) it is acceptable for blood to extend to the cisterna magna, but it cannot be centered on the cisterna magna (7, 8, 11–15).

Based on the SAH load, PNSAH can be stratified by the location of bleeding, including focal prepontine SAH, prepontine with suprasellar cistern SAH with/without intraventricular blood, and diffuse SAH (16).

### Variant

Quadrigeminal SAH can be a variant of PNSAH, which has been previously described, is characterized by blood centered in the quadrigeminal cistern (17). Quadrigeminal SAH may account for up to 20% of PNSAHs and carries a similar clinical course as PNSAH (17, 18).

### Angiographic choice

It was estimated that 7–17% of patients with ruptured posterior circulation aneurysms have a perimesencephalic pattern, and 5–7% of patients with perimesencephalic bleeding patterns will be found to have an aneurysm (19, 20). Therefore, the exclusion of aneurysms is critical to diagnose PNSAH (Figure 2). Currently, digital subtraction angiography (DSA), the gold standard for excluding intracranial aneurysm in PNSAH, is still the best choice (20–22). However, DSA is inconvenient, as it is an invasive examination in PNSAH.

Over the past decade, high-resolution CTA has become increasingly convenient and has allowed intracranial aneurysms to be diagnosed with a high sensitivity and specificity (23, 24). In recent meta-analyses of the role of CTA in PNSAH performed by Kalra et al. (25) in 2015 that included 40 studies with 1031 PNSAHs and Geng et al. (26) in 2019 that included 13 studies with 588 PNSAHs, the outcomes showed that an initial negative CTA was adequate in excluding aneurysms in PNSAH, which suggested modern high-resolution CTA is an acceptable replacement for DSA to rule out aneurysms in PNSAH (12, 27, 28).

In addition, by extending the time of image acquisition of CTA, both intracranial arteries and deep veins can be shown, and more image information can be obtained, which is helpful in PNSAH (Figures 1F, 3).

### Possible causes

The etiology of PNSAH is not completely clear. The hemorrhage origin should be focused on the region around the midbrain (Figure 3). The current possible etiology of PNSAH may include a venous origin and arterial causes. Arterial causes have been described only in case reports, such as rupture of a perforating artery, basilar artery dissection, and transient microaneurysm of the basilar artery, and they can escape angiographic detection by total disruption or spontaneous thrombosis (5). When definite arterial causes are identified, SAH should be called SAH with a perimesencephalic pattern (Figure 2).

**Figure 4:**
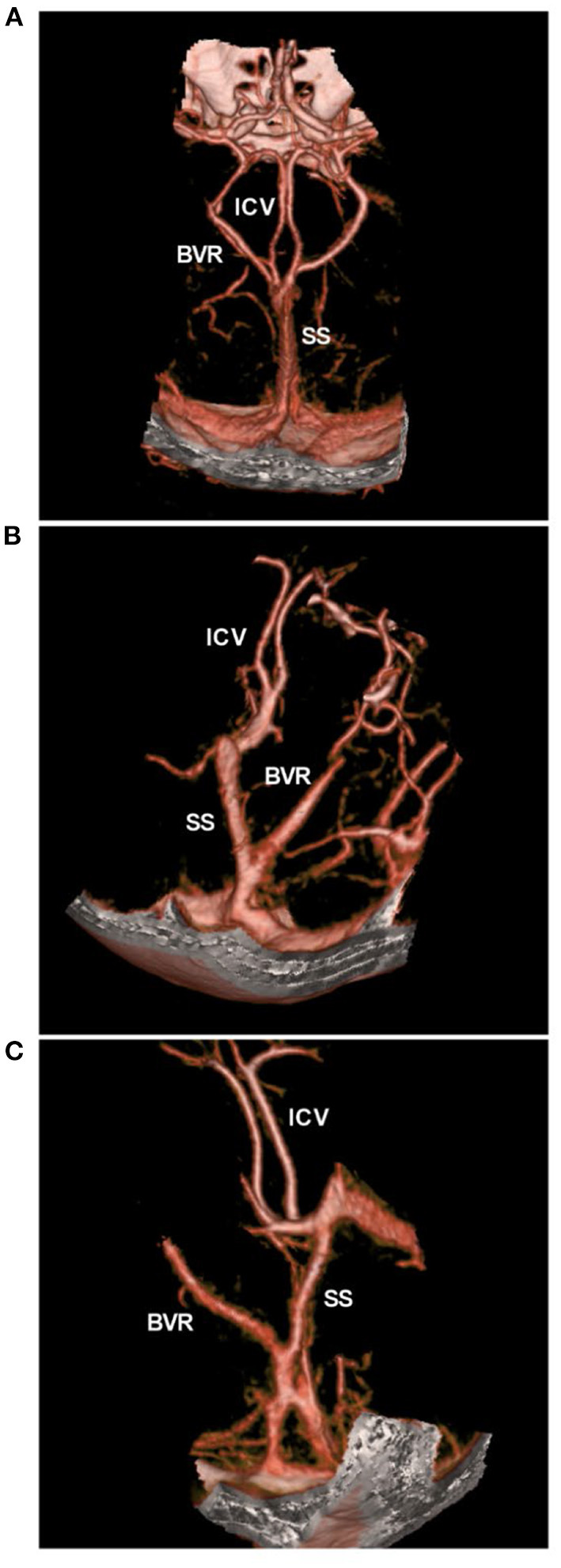
BVR variants in CTA. **(A)** CTA showing the normal pattern of deep veins, bilateral ICVs and BVRs that flow into the vein of Galen. **(B,C)** CTA showing the BVR that flowed into the straight sinus. BVR, Basal vein of Rosenthal; CTA, computed tomography angiography; ICV, internal cerebral vein, SS, straight sinus.

The current common hypothesis is that PNSAH originates from a rupture of the deep veins around the midbrain (9, 29, 30). The deep veins around the midbrain mainly include the basal vein of Rosenthal (BVR) (31). BVR has three variants: type A (normal pattern): the BVR mainly drains into the vein of Galen; type B (discontinuous pattern): the BVR drains anteriorly into the cavernous sinus and posteriorly into the vein of Galen; and type C (primitive variant): the BVR drains into veins other than the vein of Galen (29, 32). The BVR variants are shown in Figure 4.

**Figure 5:**
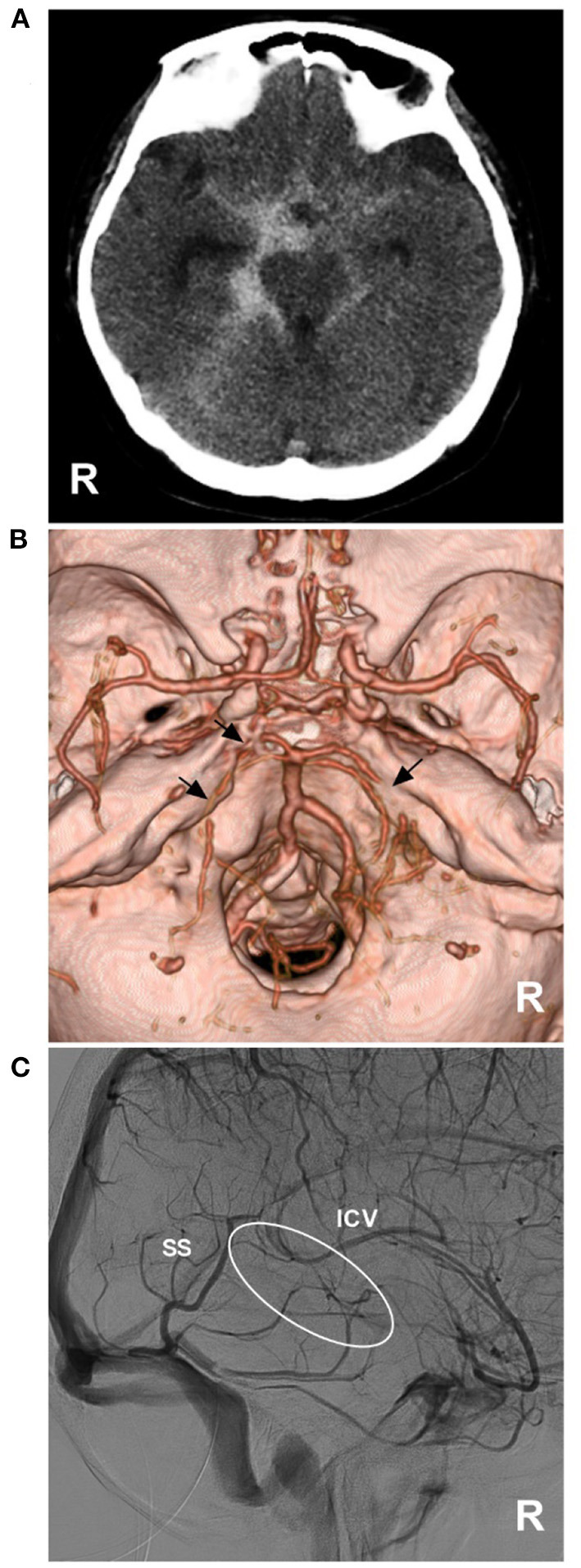
PNSAH with BVR absence. **(A)** CT showing a PNSAH that extended to the posterior part of the anterior interhemispheric fissure and the basal part of the Sylvian fissure. **(B)** CTA showing the multiple vasospasms (arrows) of the bilateral posterior cerebral arteries, without positive findings of bleeding. **(C)** DSA showing the absence of the right BVR (circle). BVR, Basal vein of Rosenthal; CT, computed tomography; CTA, computed tomography angiography; DSA, digital subtraction angiography; ICV, internal cerebral vein; PNSAH, perimesencephalic non-aneurysmal subarachnoid hemorrhage; R, right; SS, straight sinus.

In PNSAH, the incidence of type C BVR was high. In a systematic review by Rouchaud et al. (33) of 334 PNSAHs, only 18.3% of patients had bilateral normal BVR drainage, and the incidence of a primitive BVR in at least 1 hemisphere was 47.7%. A PNSAH with BVR absence is shown in Figure 5. In addition, dural sinus variations can be associated with PNSAH, including transverse sinus and superior petrosal sinus hypoplasia, inferior petrosal sinus and clival plexus hyperplasia. In Brugada-Bellsola et al.'s (34) report, more than three presentations of the above variant of the dural sinus were common in PNSAH. These abnormal anatomies may render the BVR susceptible to stretching and tearing against the tentorial edge, or the anomalies may change the draining pattern of BVR, thus leading to venous rupture (33, 34).

Deep vein rupture in PNSAH may be associated with the Valsalva maneuver, swimming, golfing, heavy lifting and gymnastics, which can produce increased intrathoracic pressure to block the internal jugular venous return, resulting in intracranial venous hypertension and leading to deep vein breakdown, which supports a venous origin of PNSAH (35). In Canneti et al.'s (36) report of spontaneous SAH with negative angiography, the Valsalva maneuver/exercise was responsible for 47.1% of the patients in the PNSAH group. However, due to the high variance of the deep veins around the midbrain, CTA and DSA still cannot confirm the evidence of a venous rupture, although a venous rupture was suspected (37).

### Pathology and pathophysiology

#### Hemorrhage in the cistern

In aneurysmal SAH, oxyhemoglobin from the arteries in brain cisterns is present at high concentration (38, 39). In PNSAH, oxyhemoglobin from the vein, which is at a low concentration, cannot trigger stress and immune-mediated inflammatory responses (40, 41). Severely increased intracranial pressure is rare at the onset of PNSAH due to the low incidence of low-pressure venous hemorrhage, which indicates that patients with PNSAH have good cerebral perfusion (42). Fragata et al.'s (43) study of CT perfusion showed that patients with PNSAH had better cerebral perfusion than aneurysmal SAH patients in the first 72 h.

#### Hydrocephalus

In PNSAH, the intraventricular blood is a result of from blood sedimentation due to cerebrospinal fluid circulation (44). Perimesencephalic cisterns filled with SAH associated with intraventricular blood can result in acute hydrocephalus due to blockage of cerebrospinal fluid at the tentorial hiatus (15, 16, 44–46). In PNSAH, the incidence of acute hydrocephalus averaged 14% (10–18%) and is usually symptomatic within 24 h (5). Persistent hydrocephalus was rare; in Mohan et al.'s (47) review, the incidence averaged 3.5% (2.3–5.1%).

#### Intracranial vasospasm

**Figure 6:**
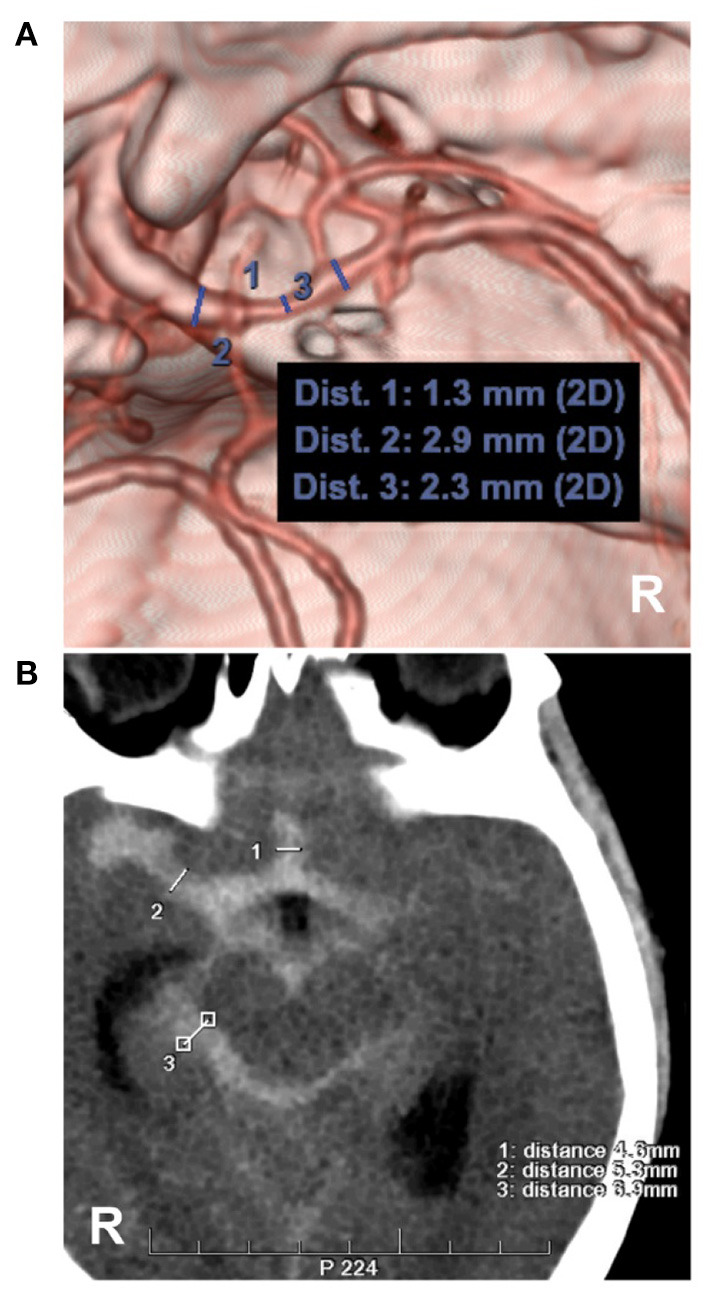
Measurements of intracranial vasospasm and SAH thickness. **(A)** CTA showing the diameter measurement of the MCA. No. 1, 2 and 3 showing the diameters of the different locations of the MCA, the location of No. 1 had a moderate vasospasm (33–66%, 1.3/2.3 and 1.3/2.9). **(B)** CT showing the measurements of the Barrow Neurological Institute scale, No. 1, 2 and 3 showing the subarachnoid hemorrhage thickness (4.6, 5.3, 6.9 mm), by the measurement across the thickest-appearing regions of the cistern or fissure. CT, computed tomography; CTA, computed tomography angiography; MCA, middle cerebral artery; R, right.

In PNSAH, the toxic effects of breakdown products of acute bleeding can result in vasospasm (48). Radiographic vasospasm was defined as any degree of vasospasm visible on either DSA or CT angiogram, and the common criterion was a reduction in the vessel caliber (Figure 6A). DSA is the gold standard for the detection of large artery vasospasm (49). Vasospasm was classified as severe if >66% vessel narrow, moderate if between 33 and 66% and mild if <33% (50, 51). In PNSAH, the incidence of vasospasm varied in different reports. The rates of radiological vasospasm were 8.3% (4.9–12.5%) in Mohan et al.'s (47) review, 9% (6–14%) in Mensing et al.'s (5) review, and 12.8% in Lee et al.'s (52) review.

Transcranial Doppler (TCD) is helpful for detecting large artery vasospasm, with a mean blood velocity of >150 cm/s or an increase in blood velocity of >50 cm/s (53, 54). Due to variable sensitivity, when using TCD to monitor and detect vasospasm, the results must be interpreted with clinical correlation (49). In Prat et al.'s (55) TCD study of PNSAH, the proximal basilar artery was most commonly involved, and vasospasm began on average on day 4, peaked on day 7, and ceased on day 15.

Most vasospasms in PNSAH are asymptomatic. Delayed ischemic neurological deficits from vasospasm were rare, as reported by Mohan et al.'s (47) review of PNSAH, the incidence was 0.7% (0–3%). However, the incidence of silent ischemia in PNSAH was high in the acute period and involved the whole brain (56). Symptomatic vasospasm was associated with the thickness of the subarachnoid blood (57–59). In 1980, Fisher et al. proposed the standard for grading SAH based on its characteristics on CT as follows: (1) no blood; (2) thin SAH <1 mm thick in vertical cisterns or fissures; (3) SAH with large clots or blood exceeding 1 mm in thickness in vertical cisterns or fissures; (4) thin or no SAH with concomitant intraventricular or intracerebral hemorrhage (57).

However, the Fisher scale categorized all thick clots as grade 3. In 2012, the Barrow Neurological Institute (BNI) scale was established by measuring the SAH thickness across the cistern or fissure (Figure 6B) as follows: (1) no blood; (2) SAH ≤5 mm thick; (3) SAH 5–10 mm thick; (4) SAH 10–15 mm thick; and 5, SAH >15 mm thick (58, 59). Grades 1–3 of the BNI scale are considered low grade. In PNSAH, a low BNI scale score is common, such as 98.6% of PNSAHs with grades 1–3 in the Walcott et al. study (46) and 98% of PNSAHs with grades 2–3 in a study by Schuss et al. (60). Therefore, PNSAH has a low incidence of symptomatic vasospasm (53).

#### Cerebral morphology

After PNSAH, neurotoxic effects from blood, reduced brain blood flow, impaired control of vascular autoregulation, and blood–brain barrier dysfunction result in encephalopathy; in long-term follow-up, focal and global cerebral atrophy can be seen (61, 62). In the Gama et al. study, patients with PNSAH had a reduction of 6.5% in whole brain volume and an increase of 21.3% in lateral ventricle volume (2). In addition, in Schweizer et al.'s (63) study, diffusion tensor imaging of MRI showed white matter injury after PNSAH.

### Clinical features and treatment

#### Clinical features

In PNSAH, 42% of patients are female; the mean age is 53 years, which is not different from patients with aneurysmal SAH (5). The risk of PNSAH is lower during the night than during the daytime hours and tends to be higher in the morning and late afternoon (64).

The clinical courses of PNSAH include acute (<3 days), subacute (4–15 days) and chronic (>15 days) symptoms (5, 65).

Acute symptoms in PNSAH are usually minimal and transient, including headache in >90% of patients, nausea and vomiting in 70% of patients, neck pain/stiffness in 24% of patients, transient focal symptoms in 9% of patients, and seizures in 1.5–5% of patients (5, 16, 66–69). Rarely, in PNSAH, dense clots in the cistern might result in cranial nerve palsy (69–71). In PNSAH, 96% of patients have a normal level of consciousness (40). Subacute symptoms may be from hydrocephalus or symptomatic vasospasm, presenting with a new focal neurological deficit (such as hemiparesis, aphasia, apraxia, hemianopia, or neglect) or a decrease of at least two points on the Glasgow Coma Scale score on the Glasgow coma scale lasting at least 1 h (49). Chronic symptoms in PNSAH mainly present as cognitive and emotional sequelae, including deficits in memory, visuospatial function, language, social cognition, executive function, processing speed, attention, depression, anxiety and mood, fear of re-bleeding, and impaired return to a previous work position (5, 61, 72–74).

#### Management

##### General management

The routine management of PNSAH can follow the recent guidelines for aneurysmal SAH, as per the American Heart Association, including monitoring of electrolyte balance, hydrocephalus, vasospasm, and seizure, as appropriate, in the intensive care unit (75). For PNSAH, patients may usually stay in the intensive care unit for 2 to 4 days until the headache improves and the acute hydrocephalus is resolved (5).

##### Hydrocephalus

**Figure 7:**
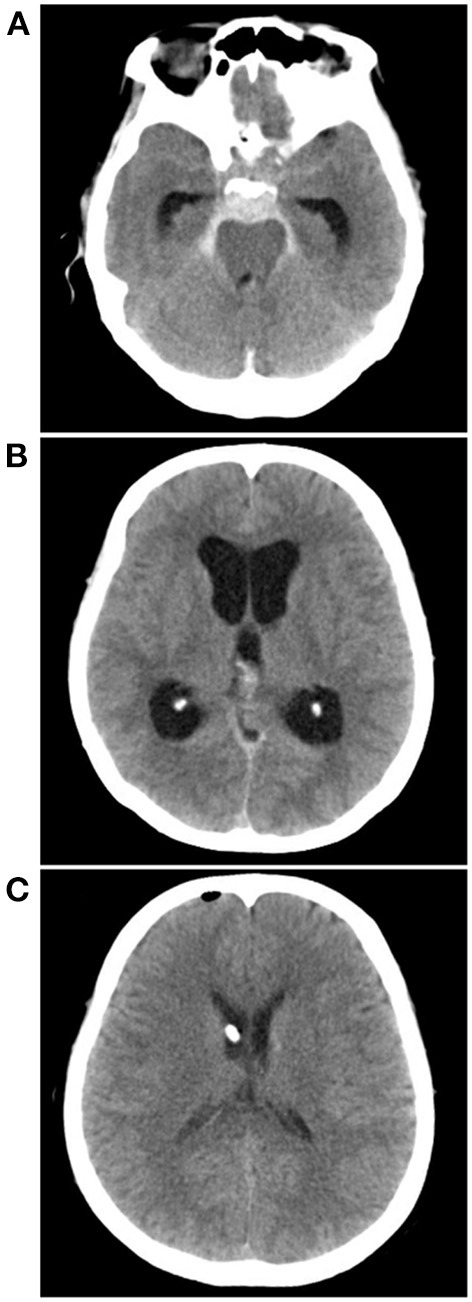
External ventricle drainage in a PNSAH. **(A)** CT showing a classic PNSAH. **(B)** CT showing acute lateral ventricle dilation. **(C)** CT showing the normal size of lateral ventricle after external ventricle drainage. CT, computed tomography; PNSAH, perimesencephalic non-aneurysmal subarachnoid hemorrhage.

In PNSAH, most acute hydrocephalus can regress spontaneously or by repeated lumbar punctures (5). External ventricle drainage in PNSAH was seldom needed (Figure 7). In Mensing et al.'s (5) review, acute hydrocephalus with a decreased level of consciousness requiring drainage occurred in only 3% of PNSAHs. Ventriculoperitoneal shunt or endoscopic third ventriculostomy may be necessary only in chronic symptomatic hydrocephalus in the minority of PNSAHs with Fisher grade 3 (53, 76–78).

##### Symptomatic vasospasm or delayed cerebral ischemia

Previously, “Tripe-H” therapy, including hypertension, hypervolemia and hemodilution, was used to prevent vasospasm (79). However, in recent years, a series of randomized controlled trials have shown that prophylactic “Triple H” therapy cannot improve SAH outcomes but increases cardiopulmonary complications, so it is not recommended in modern care practice, and the maintenance of euvolemia is sufficient (49, 79–82).

However, when symptomatic vasospasm or delayed cerebral ischemia occurs, the induction of hypertension is recommended unless blood pressure is elevated at baseline or cardiac status precludes it (49). Cerebral angioplasty and/or selective intra-arterial vasodilator therapy may be considered for patients with symptomatic vasospasm, particularly those who are not responding to hypertensive therapy, and the future is worth looking forward to (49, 83).

### Prognosis

In general, PNSAH has a benign clinical course and excellent prognosis; in long-term follow-up, death has rarely been reported (5, 84). The prognosis can be evaluated with the survival rate, daily living activity and cognitive sequelae (16).

#### Survival rate

Patients with PNSAH have a nearly normal life expectancy, and no restrictions should be imposed on these patients. In a systematic review by Wang et al. (85) patients with PNSAH showed a 13-month survival rate of 97.2% and a 30-month survival rate of 98.6%. In Greebe et al.'s (84) report of PNSAH consisting of 160 patients, with a total number of 1,213 patient-years, no new SAH occurred, and 11 patients died; however, none of the deaths were direct consequences of PNSAH, and patients have a normal life expectancy.

#### Daily living activity

A favorable outcome of daily living activity was defined as a modified Rankin Scale (mRS) of 0–2, representing functional independence (40, 86, 87). After PNSAH, the long-term activities of daily living are excellent. In Mohan et al.'s (47) review, 95.3% of patients at hospital discharge, 93.9% of patients at follow-up from 3 to 6 months, and 92.7% of patients at follow-up of more than 1 year had favorable outcomes. In Mensing et al.'s (5) review of PNSAH, 99% of patients at hospital discharge and 94–100% of patients at follow-up ranging from 0.8 to 90 months had favorable outcomes.

#### Cognitive function

Cognitive deficits may persist for a long time after PNSAH (73). Even after 7 years, some patients with PNSAH revealed problems with attentional activation and refinement speed as well as long-term non-verbal memory, and they could not return to their former jobs (88). In addition, fatigue was a problem. In De Vries et al.'s (74) study, 5 years after PNSAH, one-third of the patients still reported fatigue. Therefore, subsequent rehabilitation of patients with PNSAH is necessary (89).

#### Re-bleeding

Re-bleeding with PNSAH is uncommon. Rahme et al. (90) estimated that the chances of recurrent PNSAH would be 79/billion in a lifespan of 80 years. In a literature review by Li et al. (91) until 2021, only six previous reports described PNSAH recurrence, three of which were controversial, and the time span of recurrent PNSAH was uncertain, ranging from 5 days to 12 years. Therefore, the incidence of re-bleeding was extremely low.

### Summary

PNSAH is a distinctive disease. Current high-resolution CTA is an acceptable replacement for DSA to rule out aneurysms in PNSAH with strict criteria. The current acceptable hypothesis on the etiology of PNSAH is deep vein rupture from aberrant venous anatomy. PNSAH is often associated with mild symptoms and with a lower incidence of complications. For PNSAH, conservative treatment is mainstream. PNSAH has a benign clinical course and excellent prognosis.

## Author contributions

JY contributed to the conception and design of the manuscript and critically revised the manuscript. KH wrote the manuscript and collected the medical records of the patients. All authors approved the final version of this manuscript.

## Conflict of interest

The authors declare that the research was conducted in the absence of any commercial or financial relationships that could be construed as a potential conflict of interest.

## Publisher's note

All claims expressed in this article are solely those of the authors and do not necessarily represent those of their affiliated organizations, or those of the publisher, the editors and the reviewers. Any product that may be evaluated in this article, or claim that may be made by its manufacturer, is not guaranteed or endorsed by the publisher.
